# Genetic pathways for differentiation of the peripheral nervous system in ascidians

**DOI:** 10.1038/ncomms9719

**Published:** 2015-10-30

**Authors:** Kana Waki, Kaoru S. Imai, Yutaka Satou

**Affiliations:** 1 Department of Zoology, Graduate School of Science, Kyoto University, Kyoto 606-8502, Japan; 2 Department of Biological Sciences, Graduate School of Science, Osaka University, Toyonaka 560-0043, Japan; 3 CREST, JST, Sakyo, 4-1-8 Honcho, Kawaguchi, Saitama 332-0012, Japan

## Abstract

Ascidians belong to tunicates, the sister group of vertebrates. Peripheral nervous systems (PNSs) including epidermal sensory neurons (ESNs) in the trunk and dorsal tail regions of ascidian larvae are derived from cells adjacent to the neural plate, as in vertebrates. On the other hand, peripheral ESNs in the ventral tail region are derived from the ventral ectoderm under the control of BMP signalling, reminiscent of sensory neurons of amphioxus and protostomes. In this study, we show that two distinct mechanisms activate a common gene circuit consisting of *Msx*, *Ascl.b*, *Tox*, *Delta.b* and *Pou4* in the dorsal and ventral regions to differentiate ESNs. Our results suggest that ventral ESNs of the ascidian larva are not directly homologous to vertebrate PNSs. The dorsal ESNs might have arisen via co-option of the original PNS gene circuit to the neural plate border in an ancestral chordate.

## Abstract (web summary)

The evolutionary origin of the peripheral nervous systems (PNSs) is poorly understood. Here, the authors show that two mechanisms activate gene circuits in ascidians to differentiate epidermal sensory neurons, which suggests that vertebrate PNSs arose via cooption of the ancient PNS gene circuit.

## Graphical abstract

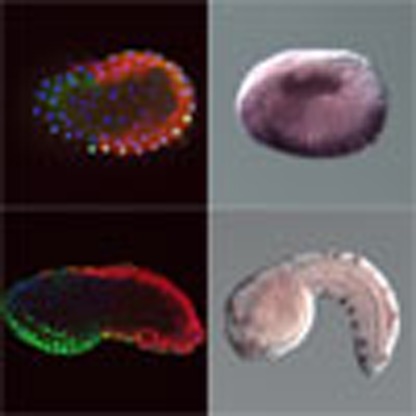

Peripheral nervous systems (PNSs) include sensory neurons. In vertebrate embryos, PNSs arise from the border of the neural plate^1^,^2^,^3^,^4^. Cranial neural crest cells and placodes contribute to the cranial PNS, whereas the caudal PNS is thought to be derived only from neural crest cells. In anamniote embryos, additional mechanosensory neurons called Rohon-Beard sensory neurons are also formed from the neural plate border^5^,^6^. On the other hand, in cephalochordates, the most basal chordate group, in which no neural crest cells or placodes have been identified, epidermal sensory neurons (ESNs) are found along the entire length of larval and adult bodies^7^,^8^. These ESNs are derived from the ventral ectodermal region of neurula embryos.

*Ciona intestinalis* is a member of the tunicates, the closest relatives of vertebrates. In this species, the palps, which contain sensory neurons and differentiate at the anterior end of the larva, are derived from the anterior border of the neural plate^9^. By contrast, the pigment cells, which differentiate in the brain, are derived from the anterolateral border of the neural plate^10^. Indeed, the anterior and anterolateral borders of the neural plate are suggested to be a rudimentary placode and primitive neural crest, respectively^11^,^12^,^13^,^14^,^15^. The posterolateral border of the neural plate gives rise to the dorsal row of the tail nerve cord and dorsal epidermis, and ESNs are differentiated from this dorsal epidermis (Supplementary Fig. 1)^16^. In addition to these PNS neurons, the ascidian larva has ESNs in the ventral region of the tail. A cell lineage analysis revealed that this ventral region is not derived from the border of the neural plate^16^.

The posterolateral border of the neural plate is induced at the 32-cell stage in a process coordinated by four signalling molecules^17^,^18^,^19^,^20^,^21^,^22^. Fgf9/16/20 signalling activates *Otx* and *Nodal* in a cell pair called b6.5 (Supplementary Fig. 1). Signalling of EfnA.d (formerly EphrinA-d, renamed according to a recently published nomenclature rule^23^), Gdf1/3-r (called Gdf1/3-like or Orphan TGFβ 2) and Admp (a bone morphogenetic protein (BMP)-related ligand) negatively regulates expression of *Otx* and *Nodal*. The Otx transcription factor and Nodal signalling cooperatively activate expression of *Msx* (formerly *Msxb*) and *Delta.b* (formerly *Delta-like* or *Delta2*)^24^,^25^. On the other hand, the ventral ESNs are induced by Admp, which is expressed in the endoderm lining the ventral ectodermal region^16^,^25^. Although the detailed molecular mechanisms remain to be elucidated, the ventral ESN lineage cells also express *Msx* and *Delta.b*^16^,^26^.

In neural induction of vertebrate embryos, BMPs negatively regulate neural fate, FGFs positively induce neural fate^27^,^28^,^29^,^30^,^31^ and neural crest and placodes arise from a region at the border of the neural plate^32^. On the other hand, in cephalochordate embryos, BMPs positively induce ESNs^33^. BMP signalling also positively regulates PNS formation in protostomes, including flies and annelids^34^,^35^. Thus, in regard to the cell lineage and signalling necessary for differentiation, the anterior PNS neurons and the dorsal PNS neurons in *Ciona* larvae are reminiscent of PNS neurons in vertebrate embryos, whereas the ventral PNS neurons are reminiscent of PNS neurons in cephalochordate and protostome embryos. Given that the innovation of neural crest cells and placodes is thought to be a key event in the course of evolution from primitive invertebrate chordates to vertebrates^32^,^36^, it is possible that the *Ciona* embryo might have both vertebrate-type and invertebrate-type PNS neurons. In the present study, we dissect a gene circuit involved in differentiation of ventral ESNs in the *Ciona* embryo, and compare it with a gene circuit involved in differentiation of dorsal ESNs for understanding evolution of PNSs in chordates.

## Results

### BMP signalling in the ventral ectodermal region

**Figure 1:**
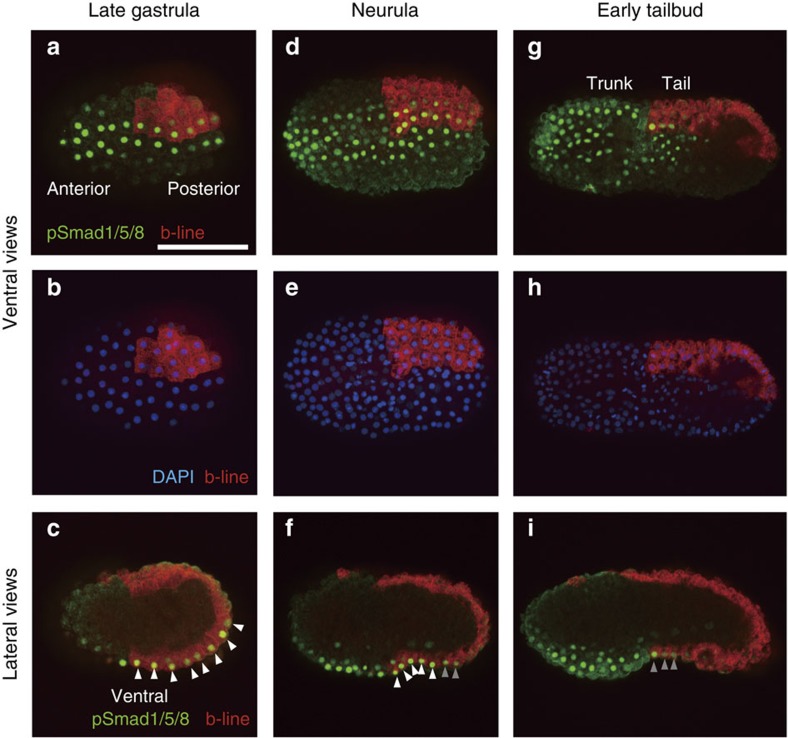
BMP signalling is active in the ventral ectoderm from the late-gastrula to the tailbud stages. One of the posterior animal cells of eight-cell embryos were labelled with DiI (red) to show the anterior–posterior boundary and left–right boundary. (**a**,**c**,**d**,**f**,**g**,**i**) BMP signalling was detected with an antibody against phosphorylated Smad1/5/8 (green). (**b**,**e**,**h**) DAPI staining shows nuclei (blue) of the embryos shown in **a**,**d** and **g**. Ventral views are shown in **a**, **b**, **d**, **e**, **g** and **h**, and lateral views are shown in **c**, **f** and **i**. Photographs are Z-projected image stacks overlaid in pseudocolour. White and grey arrowheads in **c**, **f** and **i** indicate strong and weak signals, respectively. Scale bar, 100 μm (**a**). DAPI, 4,6-diamidino-2-phenylindole.

Previous studies showed that the ventral ectodermal region of the tail is induced by a BMP ligand, Admp^16^,^25^. To determine when and where this signalling works, we used an antibody against phosphorylated Smad1/5/8 (pSmad1/5/8). To confirm that this antibody is immunoreactive against pSmad1/5/8 in ascidian embryos, we first stained early-tailbud embryos, in which BMP signalling is active in the ventral trunk region^37^. As shown in Fig. 1g, we detected strong pSmad1/5/8 staining in the ventral trunk region. In addition, when *Bmp2/4* was overexpressed in the entire epidermis using the *Dlx.b* upstream region, pSmad1/5/8 staining was detected in the entire epidermis (Supplementary Fig. 2a,c). Conversely, when Noggin, which is an antagonist for BMP ligands, was overexpressed, pSmad1/5/8 staining was barely detectable (Supplementary Fig. 2b,d). Thus, this antibody successfully detected pSmad1/5/8 in ascidian embryos.

As shown in Fig. 1a–c, we observed strong pSmad1/5/8 staining in two lines of cells of the ventral ectoderm from the anterior to the posterior end at the late-gastrula stage. We labelled one of two b4.2 blastomeres of the eight-cell embryo with DiI to identify the anterior–posterior border, because the tail ectoderm is derived from a pair of b4.2 cells. The tail region contained eight pSmad1/5/8-positive rows (Fig. 1c). At the neurula stage after the next cell division (10th division; most cells divide laterally), we detected pSmad1/5/8 in the anterior rows within the b4.2-line ectodermal cells and in the ventral ectodermal region of the trunk (Fig. 1d–f). The anterior five rows in the b4.2-line ectoderm contained a strong signal, and two or three additional rows contained a weaker signal. At the early-tailbud stage after the next cell division (11th division; most cells divide along the anterior–posterior axis), we detected a weak pSmad1/5/8 signal in only three anterior rows of the b4.2-line ectodermal cells (Fig. 1g–i).

**Figure 2:**
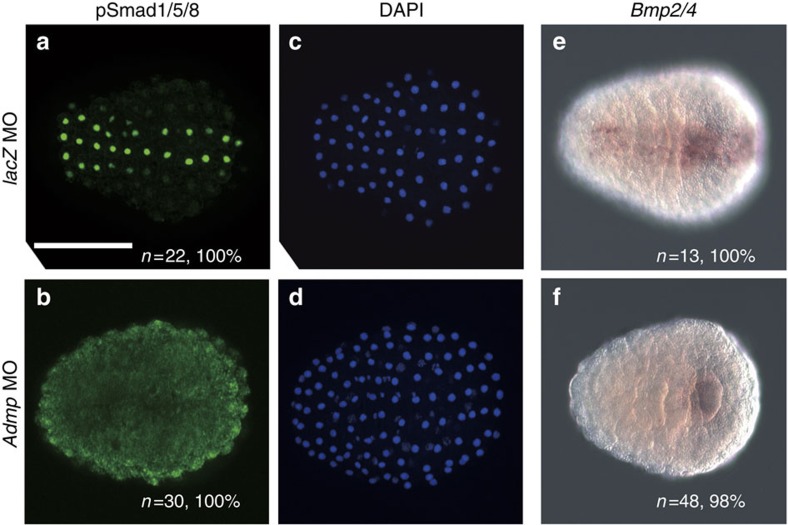
*Admp* activates Bmp signalling and *Bmp2/4* expression in the ventral ectoderm at the late-gastrula stage. (**a**,**b**) BMP signalling was detected with an antibody against phosphorylated Smad1/5/8 (green) (**a**) in an embryo injected with a control *LacZ* MO, and (**b**) in an embryo injected with an *Admp* MO. (**c**,**d**) DAPI staining shows nuclei (blue) of the embryos shown in **a** and **b**. (**e**,**f**) Expression of *Bmp2/4* at the late-gastrula stage is observed in the ventral ectoderm in an embryo injected with a control *LacZ* MO (**e**), while it is lost in an embryo injected with an *Admp* MO (**f**). Scale bar, 100 μm (**a**). The number of embryos examined and the proportion of embryos that each panel represents are shown within the panels. DAPI, 4,6-diamidino-2-phenylindole.

Indeed, pSmad1/5/8 staining at the late-gastrula stage was lost in embryos injected with a morpholino antisense oligonucleotide (MO) against *Admp* (Fig. 2b,d), while it was not affected in control embryos injected with a control MO against *Escherichia coli lacZ* (Fig. 2a,c). *Bmp2/4* is activated by Admp signalling in the ventral trunk epidermis at the tailbud stage^37^, and begins to be expressed in the ventral epidermis at the late-gastrula stage^26^. We examined whether the expression of *Bmp2/4* at the late-gastrula stage is also induced by Admp, and found that *Bmp2/4* expression was lost in *Admp* morphants (Fig. 2e,f). Because Smad1/5/8 may be phosphorylated and activated by Bmp2/4 in addition to Admp, we collectively call signalling of Bmp2/4 and Admp as BMP signalling hereafter.

### *Tbx2/3* is required for *Msx* expression in the ventral region

*Msx* is important for differentiation of the dorsal ESNs^24^,^25^, and is also expressed in the ventral tail ectoderm^26^, suggesting that *Msx* is also important for differentiation of the ventral ESNs. To confirm this possibility, we injected a MO against *Msx* into fertilized eggs. In the resultant morphant embryos, *Pou4* and *Celf3.a* (*Etr-1*), which mark ESNs^26^,^38^ (see Fig. 7c,e), were not expressed (Supplementary Fig. 3a,b) in dorsal and ventral ESNs, except in a few ESNs near the tip of the tail. Because *Msx* is not expressed in the most posterior region, it is not surprising that ESNs near the tip of the tail of *Msx* morphants expressed *Pou4* and *Celf3.a*. We also found that *Pou4* and *Celf3.a* were ectopically expressed, when *Msx* was overexpressed in the entire epidermis using the *Dlx.b* upstream region (Supplementary Fig. 3c,d).

**Figure 3:**
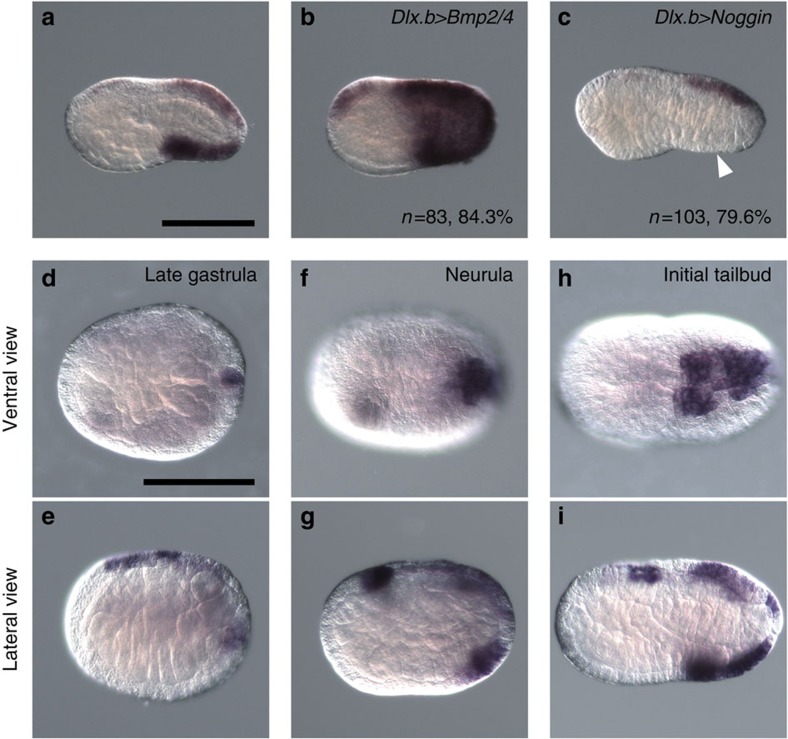
*Msx* is under the control of Bmp signalling, and begins to be expressed in the ventral tail ectoderm at the neurula stage. (**a**–**c**) *Msx* expression (**a**) in a control early-tailbud embryo, (**b**) in an embryo with *Bmp2/4* overexpression under the *Dlx.b* enhancer and (**c**) in an embryo with *Noggin* overexpression under the *Dlx.b* enhancer. The white arrowhead indicates the region in which *Msx* expression is lost. The number of embryos examined and the proportion of embryos that each panel represents are shown within the panels. (**d**–**i**) *Msx* expression (**d**,**e**) at the late-gastrula stage, (**f**,**g**) neurula stage and (**h**,**i**) initial-tailbud stage. (**d**,**f**,**h**) Ventral views and (**e**,**g**,**i**) lateral views are shown. Scale bar, 100 μm (**a**,**d**).

A previous study showed that expression of *Celf3.a* in the ventral ESNs is under the control of BMP signalling^16^. In the present study, we found that expression of *Msx* in ventral tail ectoderm was similarly downstream of BMP signalling, because *Msx* expression in ventral tail ectoderm was upregulated in embryos overexpressed with *Bmp2/4* using the *Dlx.b* enhancer, and downregulated in embryos overexpressed with *Noggin* (Fig. 3a–c).

We next investigated whether *Msx* began to be expressed at the same stage as *Bmp2/4*, which is activated by Admp signalling, as described above. Although the expression pattern of *Msx* has been previously described^26^,^39^, we again examined *Msx* expression to determine the exact timing of its expression in ventral ectodermal cells (Fig. 3d–i). *Msx* was not expressed in the ventral ectoderm at the late-gastrula stage, and began to be expressed in the posterior half of the ventral tail ectoderm at the neurula stage. At the initial-tailbud stage, *Msx* was expressed in the entire ventral region of the tail ectoderm. Thus, because its expression initiates later than that of *Bmp2/4*, *Msx* is not likely to be a direct target of Admp signalling.

**Figure 4:**
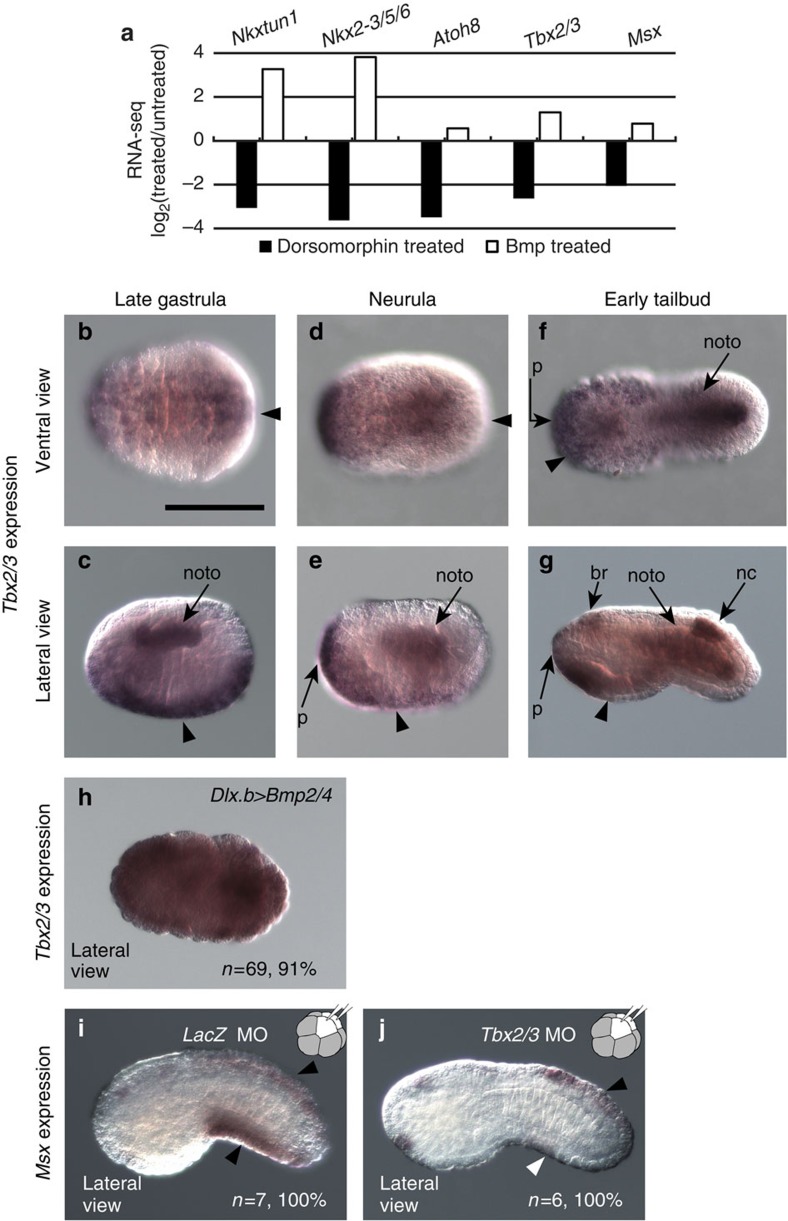
*Tbx2/3* is expressed under Bmp signalling and regulates *Msx* expression in the ventral ectoderm. (**a**) RNA-seq experiments identified five transcription factor genes greatly upregulated and downregulated in embryos treated with Bmp4 and dorsomorphin, respectively (*P* values adjusted for multiple testing <0.01, which were calculated by DEseq^52^; numbers of sequencing tags for untreated control embryos, Bmp4-treated embryos and dorsomorphin-treated embryos were 17,408,671, 10,657,653 and 16,157,915, respectively). (**b**–**g**) *Tbx2/3* expression (**b**,**c**) at the late-gastrula stage, (**d**,**e**) neurula stage and (**f**,**g**) early-tailbud stage. Expression in the ventral ectoderm is indicated by arrowheads. Note that *Tbx2/3* is also expressed in the notochord (noto), nerve cord (nc), brain (br) and palps (p). (**h**) *Tbx2/3* expression is expanded throughout the entire epidermal region of embryos with *Bmp2/4* overexpression under the control of the upstream sequence of *Dlx.b*. (**i**,**j**) MOs against (**i**) *LacZ* and (**j**) *Tbx2/3* were injected into the left and right posterior animal cells (b4.2) of eight-cell embryos. While *Msx* expression was not affected (**i**) in embryos injected with the *LacZ* MO and (**j**) in the dorsal region of *Tbx2/3* morphant embryos (black arrowheads), it was greatly reduced in the ventral region of *Tbx2/3* morphant embryos (white arrowhead). Scale bar, 100 μm (**b**). The number of embryos examined and the proportion of embryos that each panel represents are shown in **h**–**j**. All embryos are shown with anterior to the left.

To identify candidate genes that are directly regulated by Admp signalling and regulate *Msx* expression, we used RNA sequencing (RNA-seq) to compare gene expression in late-gastrula embryos treated with recombinant BMP4 or a BMP inhibitor, dorsomorphin. Five transcription factor genes, including *Msx*, were greatly upregulated in embryos treated with BMP4 and downregulated in embryos treated with dorsomorphin (Fig. 4a). *Nkxtun1* (formerly *Nkx-A*) and *NK2-3/5/6* (formerly *NK4*) are expressed specifically in ventral epidermis of the trunk at the tailbud stage, whereas *Atoh8* (formerly *Net*) is not specifically expressed in the ventral epidermis^26^. On the other hand, *Tbx2/3* is expressed in the ventral ectoderm of the tail^26^,^40^,^41^. We therefore determined the exact timing of the initiation and termination of *Tbx2/3* expression (Fig. 4b–g). At the late-gastrula stage, *Tbx2/3* was expressed in the entire ventral ectoderm (Fig. 4b,c). At the neurula stage, expression of *Tbx2/3* was diminished in the posterior half of the tail ectoderm, and became weaker in the rest of the ventral ectoderm (Fig. 4d,e). At the early-tailbud stage, expression of *Tbx2/3* vanished completely in the ventral tail ectoderm (Fig. 4f,g). Thus, expression of *Tbx2/3* preceded *Msx* expression. *Tbx2/3* was indeed activated by Bmp signalling, as demonstrated by the observation that it was ectopically expressed in *Bmp2/4*-overexpressing embryos (Fig. 4h).

Next, we injected a *Tbx2/3* MO into both of the left and right b4.2 blastomeres at the eight-cell stage to confirm that *Tbx2/3* regulated *Msx* in the tail ectoderm cell-autonomously^26^ (we did not injected the MO into fertilized eggs, because *Tbx2/3* is also expressed in non-b-line cells). In these *Tbx2/3* morphants, *Msx* expression was downregulated in the ventral tail ectoderm of tailbud embryos, but was unaffected in the dorsal tail ectoderm (Fig. 4i,j). For a further confirmation, the *Tbx2/3* MO was injected into one of the posterior animal cells of eight-cell embryos (b4.2), *Msx* expression was lost in the ventral region on the injected side (Supplementary Fig. 4a,b). Thus, BMP signalling activates *Tbx2/3* in the ventral tail ectoderm at the late-gastrula stage, and *Tbx2/3* is required for activating *Msx*.

Although *Tbx2/3* is required for *Msx* expression in the ventral tail ectoderm, it might be insufficient for *Msx* expression. This is because *Tbx2/3* messenger RNA (mRNA) injection into fertilized eggs failed to evoke ectopic expression of *Msx* (Supplementary Fig. 5). BMP signalling itself or unidentified factors downstream of BMP signalling, together with *Tbx2/3*, might work to activate *Msx*.

### *Ascl.b* and *Tox* are activated by *Msx* in the tail ectoderm

Because *Ascl.b* (achaete-scute family bHLH transcription factor-b; formerly *Ash2*) and *Tox* (*TOX* high-mobility group box family member; formerly *CAGF9*) are activated downstream of *Msx* in the dorsal ectoderm, where dorsal ESNs differentiate^25^, and because these two genes are also expressed in ventral tail ectoderm^26^, we investigated the possibility that *Msx* regulates the expression of these two genes in the ventral tail ectoderm.

**Figure 5:**
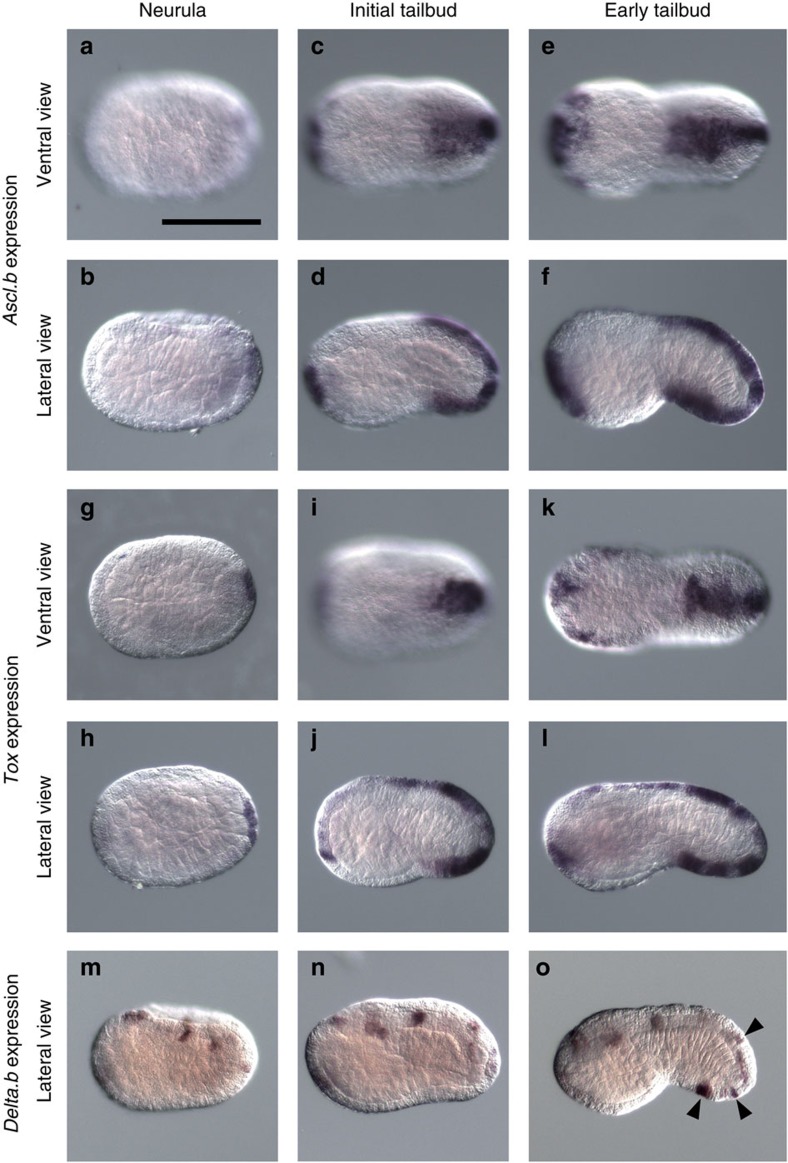
*Ascl.b* and *Tox* expression begins at the initial-tailbud stage and precedes *Delta.b* expression. (**a**–**h**) Expression of (**a**–**f**) *Ascl.b*, (**g**–**l**) *Tox* and (**m**–**o**) *Delta.b*. (**a**,**b**,**g**,**h**,**m**) at the neurula stage, and (**c**,**d**,**i**,**j**,**n**) initial-tailbud stage, and (**e**,**f**,**k**,**l**,**o**) early-tailbud stage. (**a**,**c**,**e**,**g**,**i**,**k**) Ventral views and (**b**,**d**,**f**,**h**,**j**,**l**–**o**) lateral views are shown. Prospective ESNs are indicated by black arrowheads in **o**. Scale bar, 100 μm (**a**).

Expression of these two genes in the ventral tail ectoderm was not detectable at the late-gastrula or neurula stage, and began at the tailbud stage in normal embryos (Fig. 5a–l). These genes were strongly expressed in the ventral tail ectoderm, except for several cells near the trunk–tail border at the initial-tailbud stage. At the early-tailbud stage, both genes began to be expressed throughout the tail ventral ectoderm. Thus, expression of *Ascl.b* and *Tox* began later than *Msx* expression.

**Figure 6:**
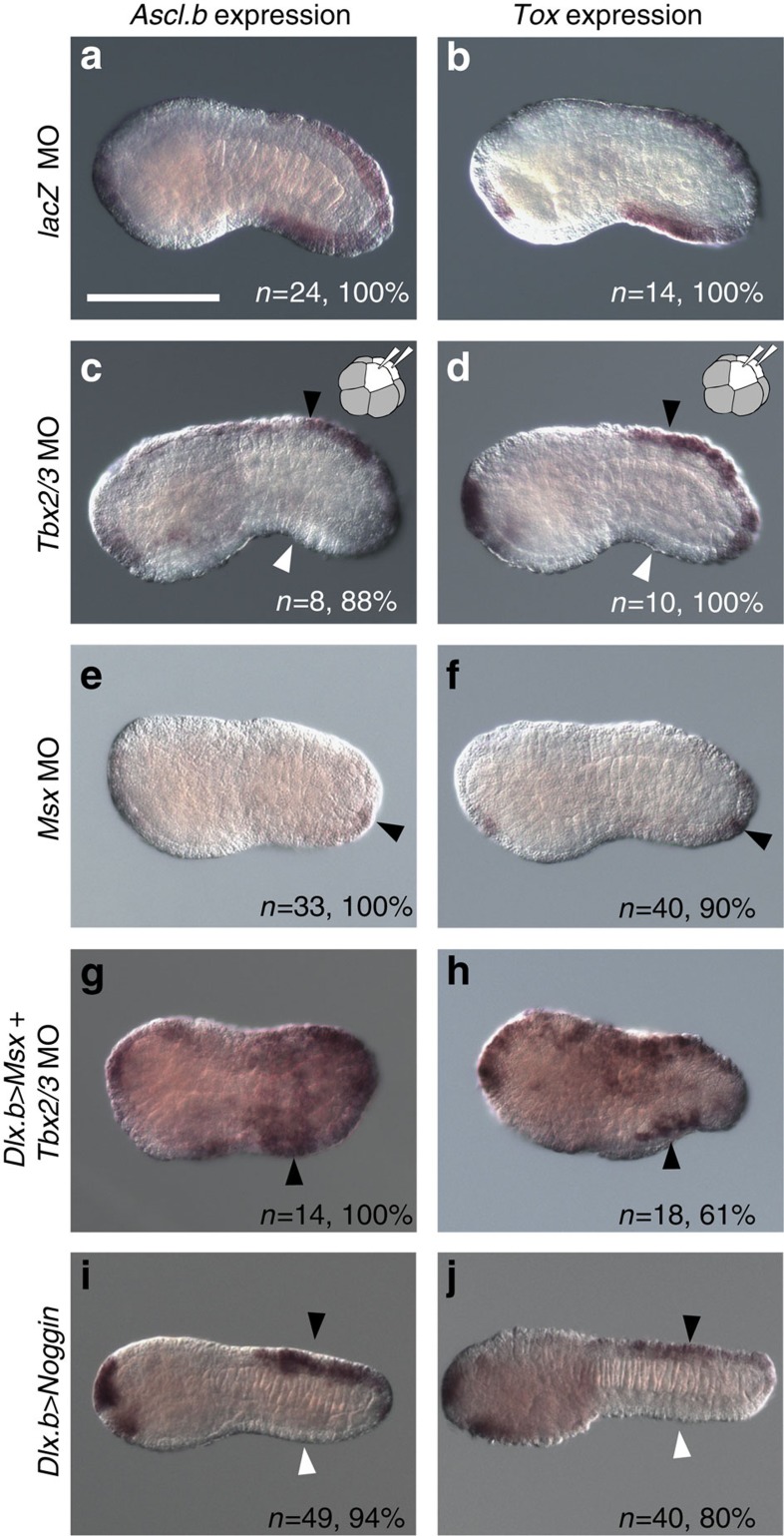
*Ascl.b* and *Tox* are under the control of *Tbx2/3*, *Msx* and Bmp signalling. (**a**,**b**) Injection of the control *LacZ* MO into fertilized eggs did not affect the expression of (**a**) *Ascl.b* or (**b**) *Tox*. (**c**,**d**) When the *Tbx2/3* MO was injected into the left and right posterior animal cells (b4.2) of eight-cell embryos, the expression of (**c**) *Ascl.b* and (**d**) *Tox* was greatly reduced in the ventral region (white arrowhead), but not in the dorsal region (black arrowhead). (**e**,**f**) The expression of (**e**) *Ascl.b* and (**f**) *Tox* in both the ventral and dorsal regions was lost in *Msx* morphants, except for a few cells in the posterior-most region (black arrowheads). (**g**,**h**) When the *Tbx2/3* MO was injected into the left and right posterior animal cells (b4.2) of eight-cell embryos after electroporation of an overexpression construct for *Msx*, the expression of (**g**) *Ascl.b* and (**h**) *Tox* was observed in the ventral region (arrowheads) and the lateral epidermal region. With this construct, *Msx* was overexpressed in the entire epidermis under the control of the upstream sequence of *Dlx.b*. Note that not all epidermal cells overexpress *Msx* because of mosaic incorporation of the electroporated plasmid. (**i**,**j**) The expression of (**i**) *Ascl.b* and (**j**) *Tox* was lost in the ventral region (white arrowheads), but not in the dorsal region (black arrowheads), of embryos with Noggin overexpression under the control of the upstream sequence of *Dlx.b*. The number of embryos examined and the proportion of embryos that each panel represents are shown. Scale bar, 100 μm (**a**).

We first confirmed that injection of the control MO did not affect expression of *Ascl.b* and *Tox* (Fig. 6a,b). Next, we injected a MO against *Tbx2/3* into both of the left and right b4.2 blastomeres. In the resultant morphant embryos, expression of *Ascl.b* and *Tox* in the tail ventral ectoderm was greatly reduced, whereas expression of the two genes in the dorsal ectoderm, in which *Tbx2/3* was not expressed, was not affected (Fig. 6c,d). Likewise, the *Tbx2/3* MO was injected into one of the posterior animal cells of eight-cell embryos (b4.2); expression of *Ascl.b* and *Tox* was lost in the ventral region on the injected side (Supplementary Fig. 4c–f). In *Msx* morphants, the expression of *Ascl.b* and *Tox* in the dorsal and ventral ectoderm, except for a few cells near the tip of the tail, was also lost (Fig. 6e,f). Thus, the expression of *Ascl.b* and *Tox* in the dorsal ectoderm is under the control of *Msx* but not *Tbx2/3*, while the expression in ventral ectoderm is under the control of *Tbx2/3* and *Msx*.

We also found that the expression of *Ascl.b* and *Tox* was recovered in the tail ventral ectoderm and observed ectopically in the head and tail ectoderm, when the *Msx* overexpression construct was introduced into fertilized eggs by electroporation and then the *Tbx2/3* MO was injected into both of the left and right b4.2 blastomeres at the eight-cell stage (Fig. 6g,h). Therefore, *Msx* can activate *Ascl.b* and *Tox* in the absence of *Tbx2/3*, and *Tbx2/3* controls *Ascl.b* and *Tox* through activation of *Msx.*

Finally, we examined the expression of *Ascl.b* and *Tox* in embryos with *Noggin* overexpression using the *Dlx.b* enhancer. In these embryos, the expression in the ventral tail ectoderm was specifically lost (Fig. 6i,j). This observation confirmed that BMP signalling is required for genes activated in the ventral tail ectoderm.

### *Tox* and *Ascl.b* are required for differentiation of ESNs

**Figure 7:**
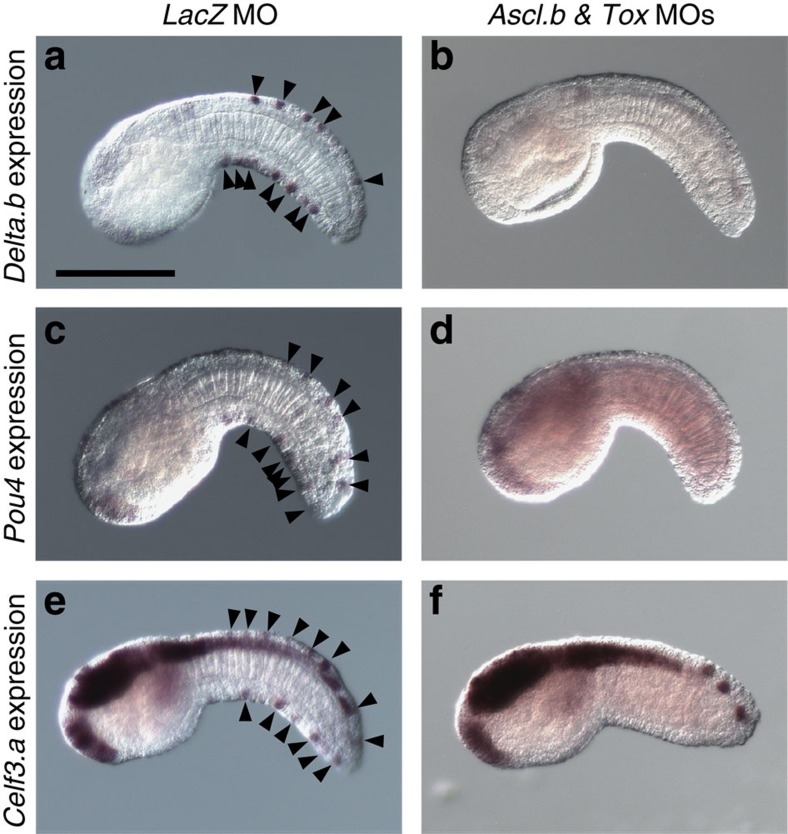
*Ascl.b* and *Tox* regulate *Delta.b*, *Pou4* and *Celf3.a*, which mark ESNs. Expression of (**a**,**b**) *Delta.b*, (**c**,**d**) *Pou4* and (**e**,**f**) *Celf3.a* marks the ESNs (black arrowheads) (**a**,**c**,**e**) in control embryos injected with the control *LacZ* MO, but is completely lost (**b**,**d**,**f**) in double morphants of *Ascl.b* and *Tox*. Lateral views are shown. Note that three spots seen near the tip of the tail in **f** are cells of the nerve cord, but not ESNs.

*Delta.b* is expressed in dorsal and ventral ESNs, and *Delta.b* signalling represses *Pou4* and *Celf3.a* (*Etr-1*) in the surrounding epidermal cells^16^,^24^,^42^. *Delta.b* expression in the ventral tail ectoderm begins in scattered cells of the posterior region and subsequently expands into the anterior tail region^16^. Through Delta/Notch–mediated lateral inhibition, the number of neurons are thought to be controlled^16^. We first investigated whether expression of *Ascl.b* and *Tox* preceded *Delta.b* expression in dorsal and ventral ESNs. *Delta.b* was not expressed in prospective ESNs at the neurula and initial-tailbud stages (Fig. 5m,n), and began to be expressed in prospective ESNs at the early-tailbud stage in the posterior region (Fig. 5o). At the middle-tailbud stage, prospective ESNs in the anterior tail region began to express *Delta.b*^16^ (see also Fig. 7a). Thus, initiation of *Delta.b* expression in prospective ESNs occurred one step later than initiation of *Ascl.b* and *Tox* expression.

When we injected MOs against either of *Ascl.b* or *Tox*, expression of *Delta.b*, *Pou4* and *Celf3.a* was lost or reduced in most embryos (Table 1; Supplementary Fig. 6). In double morphants of *Ascl.b* and *Tox*, the expression of these three genes was almost completely lost (Table 1; Fig. 7a–f). Thus, *Ascl.b* and *Tox* are both required for differentiation of ESNs in the ventral and dorsal ectoderm.

## Discussion

A previous study reported that there are two morphologically distinct populations among the ESNs of the ventral tail ectoderm, because posterior ESNs have longer axons than anterior ESNs^43^. Our analysis showed that ESNs in the anterior and posterior halves of the ventral tail ectoderm use the same gene circuit beginning with BMP signalling, although timing of the initiation of *Msx* expression differs between the anterior and posterior populations.

We showed that *Tbx2/3* is required for *Msx* expression in the ventral ectoderm. Although Tbx2/3 and its orthologues, Tbx2 and Tbx3, often act as a repressor, Tbx2 and Tbx3 are also known to act as activators^44^,^45^. Therefore, it is not surprising that Tbx2/3 directly activates *Msx* in the ventral ectoderm, although it is also possible that Tbx2/3 acts as a repressor and indirectly activates *Msx*.

**Figure 8:**
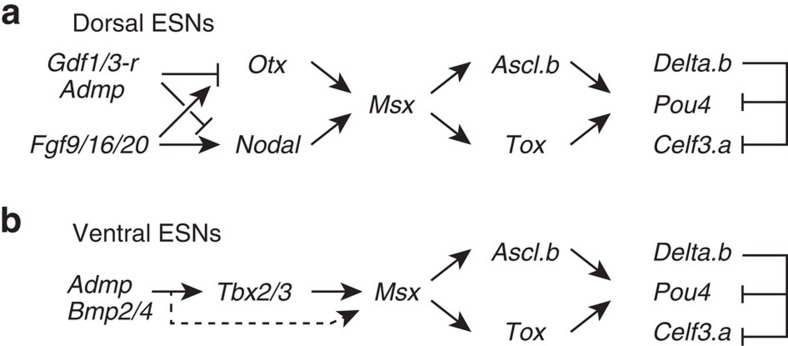
Summary of the regulatory gene circuits involved in differentiation of the dorsal and ventral ESNs. (**a**) Dorsal ESNs and (**b**) ventral ESNs share a gene circuit downstream of *Msx*, whereas the regulatory mechanisms upstream of *Msx* differ between these two lineages. These circuits are based on the results of the present and previous studies^16^,^24^,^25^,^43^. Because *Tbx2/3* alone failed to activate *Msx*, BMP signalling itself or unidentified factors downstream of BMP signalling, together with *Tbx2/3*, might work to activate *Msx* (a dotted arrow).

We also showed that ventral and dorsal tail ESNs are specified by a common genetic pathway comprising *Msx*, *Tox*/*Ascl.b* and *Delta.b*/*Celf3.a*/*Pou4*, whereas the upstream mechanism of *Msx* regulation differs between these two lineages (Fig. 8). In the dorsal lineage, *Otx* and *Nodal* are required for *Msx* expression^24^,^25^, and these two genes are activated by a combination of the activating influence of FGF signalling and the repressive influence of Ephrin, Admp and Gdf1/3-r signalling at the 32-cell stage^17^,^18^,^19^,^20^,^21^,^22^. In the ventral lineage, BMP signalling induces *Tbx2/3* at the late-gastrula stage, and this *Tbx2/3* expression is required for *Msx* expression. Hence, the evidence that co-option of the gene circuit downstream of *Msx* created a novel lineage of ESNs is persuasive. If so, which of the lineages represents the original one? We favour the hypothesis that the ventral lineage is the original one, for two reasons. First, the ventral lineage is induced by BMP signalling, and PNS neurons in amphioxus and protostomes are also induced by BMP signalling^33^,^34^,^35^. Second, although cells giving rise to the dorsal ESNs in the ascidian embryos are embedded in the epidermis and not migratory, the dorsal lineage is derived from cells located at the border of the epidermal lineage and neural plate, reminiscent of the situation in vertebrate PNSs.

In anamniote embryos, Rohon-Beard mechanosensory neurons are also formed from the neural plate border^5^. An anatomical study suggested that Retzius bipolar cells of amphioxus are homologous to Rohon-Beard sensory neurons^46^. It is possible that the ascidian dorsal ESNs are homologous to Rohon-Beard neurons, and this possibility is not necessarily mutually exclusive with the above possibility that the ascidian dorsal ESNs are homologous to vertebrate PNS neurons derived from neural crest cells. Indeed, the gene networks that specify Rohon-Beard neurons and the neural crest are overlapped considerably^47^. None of these scenarios is inconsistent with our hypothesis that a novel lineage of ESNs were born by co-option of the gene circuit downstream of *Msx*.

In vertebrate embryos, BMP signalling negatively regulates neural fate during the initial process of neural induction, but later an intermediate level of BMP signalling is required for neural crest formation^48^,^49^. *Msx* is indeed a direct target of BMP signalling at the neural plate border^50^. However, because *Msx* may not be a direct target of BMP signalling in the ventral ectoderm of the ascidian embryo, the gene circuit comprising BMP signalling, *Tbx2/3* and *Msx* in the ascidian embryo is not likely directly relevant with the gene circuit that activates *Msx* in vertebrate embryos. Taken together, the available data suggest that a common ancestor of *Ciona* and vertebrates acquired the secondary PNS lineage around the neural plate border by co-option of a gene circuit activated by *Msx*. Thus, the ventral ESNs of the ascidian larva may not be directly homologous to vertebrate PNSs, and the original lineage might have been lost in the vertebrates.

## Methods

### *In situ *hybridization and immunostaining

*C. intestinalis* adults were obtained from the National Bio-Resource Project for *Ciona*. Complementary DNA clones were obtained from our clone collection used for obtaining expressed sequence tags (ESTs)^51^. For whole-mount *in situ* hybridization, digoxigenin-RNA probes were synthesized by *in vitro* transcription with T7 RNA polymerase. Embryos were fixed in 4% paraformaldehyde in 0.1 M MOPS-NaOH (pH 7.5) and 0.5 M NaCl at 4 °C overnight and then stored in 80% ethanol. After a wash with PBS containing 0.1% tween 20 (PBST), embryos were treated with 2 μg ml^−1^ Protenase K for 30 min at 37 °C, washed again with PBST and fixed with 4% paraformaldehyde for 1 h at room temperature. Embryos were then incubated in 6 × saline sodium citrate buffer, 50% formamide, 5 × Denhardt's solution, 100 μg ml^−1^ yeast tRNA and 0.1% tween 20 for 1 h at 50 °C. After this prehybridization step, specific RNA probes were added and incubated for 16 h at 50 °C. Embryos were treated with RNase A, and incubated in 0.5 × saline sodium citrate buffer, 50% formamide and 0.1% tween 20 for 15 min at 50 °C twice. Embryos were further incubated in 0.5% blocking reagent (Roche) in PBST for 30 min, and then in 1:2,000 alkaline-phosphatase-conjugated anti-digoxigenin antibody (Roche). After a PBST wash, embryos were further washed with 0.1 M NaCl, 50 mM MgCl_2_ and 0.1 M Tris-HCl (pH 9.5), and then p-nitroblue tetrazolium chloride (NBT) and 5-bromo-4-chloro-3-indolyl phosphate (BCIP) were used for detection.

To detect activation of the Bmp signalling pathway, embryos were fixed with 3.7% formaldehyde in PBS, treated with 3% H_2_O_2_ for 30 min and then incubated with a rabbit polyclonal antibody against a synthetic phosphopeptide derived from human Smad1 (Abcam, ab97689; 1:1,000; *Ciona* Smad1/5/8 has the same amino-acid sequence in its C-terminal end) in Can-Get-Signal-Immunostain Solution B (Toyobo). The signal was visualized with a TSA kit (Invitrogen) using horseradish peroxidase-conjugated goat anti-rabbit immunoglobulin-G and Alexa Fluor 488 tyramide. For RNA-seq experiments, embryos were treated with human BMP4 (100 ng ml^−1^; HumanZyme) and dorsomorphin (100 μM; Wako) continuously after fertilization, and collected at the late-gastrula stage. RNA-seq experiments were performed using the Illumina TruSeq RNA Sample Preparation kit and the Illumina Hiseq2500 sequencer. We did not take duplicates, mainly because we used this experiment for screening, and the obtained result was confirmed with other methods. Sequence data are deposited in the SRA database under the accession numbers, DRR033088–DRR033090. DEseq was used to identify differentially expressed genes^52^.

Identifiers for genes examined in the present study are as follows: CG.KH2012.C2.421 for *Admp*, CG.KH2012.C4.125 for *Bmp2/4*, CG.KH2012.C12.562 for *Noggin*, CG.KH2012.L96.87 for *Tbx2/3*, CG.KH2012.C2.957 for *Msx*, CG.KH2012.C12.577 for *Nkxtun.a* (*Nkx-A*), CG.KH2012.C8.482 for *Nkx2-3/5/6* (*NK4*), CG.KH2012.C9.872 for *Atho8* (*Net*), CG.KH2012.L9.13 for *Ascl.b*, CG.KH2012.C3.330 for *Tox*, CG.KH2012.L50.6 for *Delta.b*, CG.KH2012.C2.42 for *Pou4* and CG.KH2012.C6.128 for *Celf3.a*.

### Gene knockdown and overexpression

The MOs (Gene Tools, LLC) against *LacZ*, *Admp*, *Tbx2/3* and *Msx*, which block translation, were used previously^25^,^53^; *LacZ*, 5′-TACGCTTCTTCTTTGGAGCAGTCAT-3′; *Admp*, 5′-TATCGTGTAGTTTGCTTTCTATATA-3′; *Tbx2/3*, 5′-GAGGTCCACACCAACACTTTAACAT-3′; *Msx*, 5′-ATTCGTTTACTGTCATTTTTAATTT-3′. We designed an additional MO against *Tbx2/3* (5′-GGTCTTCGCTATCGGTCAAACACAT-3′). Because two *Tbx2/3* MOs yielded the same phenotype (downregulation of *Msx*; Supplementary Fig. 7a), only results obtained with the first MO are shown, unless otherwise noted. We also designed one MO that blocks translation (5′-CATCGTCCATCATAAGTTGTAGCAT-3′) and one MO that blocks splicing of *Tox* (5′-TGTCCCTGGAGAATCGGCGAATCAA-3′). Because both MOs yielded the same phenotype (downregulation of *Pou4* and *Celf3.a*; Supplementary Fig. 7b,c), only results obtained with the first MO are shown, unless otherwise noted. Although we designed two MOs that block translation of *Ascl.b* (5′-GCGGTTCGTCACTTCCGGTCGCCAT-3′ and 5′-TCACTTCCGGTCGCCATTTTTGTTC-3′), the second one yielded a nonspecific phenotype; embryos injected with the second MO started to be disorganized at the eight-cell stage. However, we believe that the data obtained with the first MO are specific, because overexpression of *Ascl.b* gave opposite phenotypes (ectopic expression of *Pou4* and *Celf3.a*; Supplementary Fig. 7d,e), because *Gfp* gene, in which the first codon (ATG) was replaced with a nucleotide sequence including an *Ascl.b* MO target site (5′-ATGGCGACCGGAAGTGACGAACCGCGG-3′), was not expressed by coinjection with the *Ascl.b* MO (Supplementary Fig. 7f,g), and because phenotypes of double morphants of *Ascl.b* and *Tox* were stronger than those of single morphants of either of *Ascl.b* or *Tox*. MOs were introduced by microinjection under a microscope. All experiments were repeated at least twice with different batches of embryos.

The DNA constructs used for overexpression of *Msx*, *Bmp2/4* and *Noggin* under the *Dlx.b* upstream sequence (Ciinte.REG.KH.C7.630497–632996|*Dlx.b*) were used previously^37^. These DNA constructs were introduced by electroporation. For mRNA injection experiments, *in vitro* synthesized capped mRNA for *Tbx2/3* was prepared using an mMESSAGE mMACHINE T3 Transcription kit (Life Technologies). All experiments were repeated at least twice with different batches of embryos.

## Additional information

**Accession codes:** RNA-seq data generated in this study have been deposited in the SRA database under the accession codes DRR033088–DRR033090.

**How to cite this article:** Waki, K. *et al.* Genetic pathways for differentiation of the peripheral nervous system in ascidians. *Nat. Commun.* 6:8719 doi: 10.1038/ncomms9719 (2015).

## Supplementary Material

Supplementary Information Supplementary Figures 1-7

## Figures and Tables

**Table 1:** Effects of knockdown of *Ascl.b* and *Tox*.

**Marker genes examined**	**Morpholinos**	**Embryos not expressing the designated marker in prospective ESNs**	**Embryos expressing the designated marker in one prospective ESN**	Embryos expressing the designated marker in a reduced number of prospective ESNs*	Embryos expressing the designated marker as in wild-type embryos*
*Delta.b*	*LacZ*	0	0	0	8 (100%)
	*Ascl.b*	20 (57%)	13 (37%)	0	2 (6%)
	*Tox*	24 (29%)	20 (25%)	30 (37%)	7 (9%)
	*Ascl.b* and *Tox*	27 (100%)	0	0	0
*Pou4*	*LacZ*	0	0	1 (9%)	11 (91%)
	*Ascl.b*	33 (91%)	1 (3%)	2 (6%)	0
	*Tox*	8 (32%)	2 (8%)	13 (52%)	2 (8%)
	*Ascl.b* and *Tox*	26 (86%)	2 (7%)	0	2 (7%)
*Celf3.a*	*LacZ*	0	0	0	12 (100%)
	*Ascl.b*	24 (83%)	4 (14%)	1 (3%)	0
	*Tox*	21 (44%)	15 (31%)	12 (25%)	0
	*Ascl.b* and *Tox*	26 (90%)	2 (7%)	0	1 (3%)

ESN, epidermal sensory neuron.

^*^Because the number of ESNs varies between individuals^43^, we counted the number of prospective ESNs expressing *Pou4* in 63 unperturbed embryos. Because the minimum number of prospective ESNs was seven, embryos with two to six cells expressing either of the markers were considered to have a reduced number of ESNs, whereas embryos with seven or more cells expressing either of the markers were considered to be normal.
