# Supplementary material for: Genetic pathways for differentiation of the peripheral nervous system in ascidians
Source: Nat Commun. 2015 Oct 30;6:8719. doi: 10.1038/ncomms9719 (PMC4640076; doi:10.1038/ncomms9719)
Supplement: Supplementary Information — Supplementary Figures 1-7 [file ncomms9719-s1.pdf]

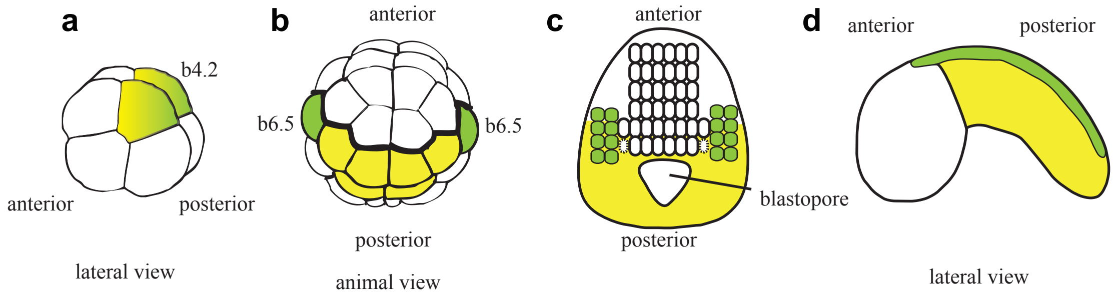

Supplementary Figure 1. Illustrations showing the b-line ectodermal lineage. (a) Colored cells indicate the posterior animal cells (b4.2) at the 8-cell stage, which contribute to the nerve cord and epidermal cells in the tail. (b-d) The b6.5 lineage is colored in green, and the remaining b-line lineage is colored in yellow in embryos at (b) the 32-cell stage, (c) late gastrula stage, and (d) early tailbud stage. These cells are derived from b4.2 of 8-cell embryos. White boxes in (c) indicate cells in the neural plate that are not derived from the b-line lineage. The b6.5 lineage cells contribute to the nerve cord, dorsal epidermis and epidermal sensory neurons within the dorsal epidermal territory.

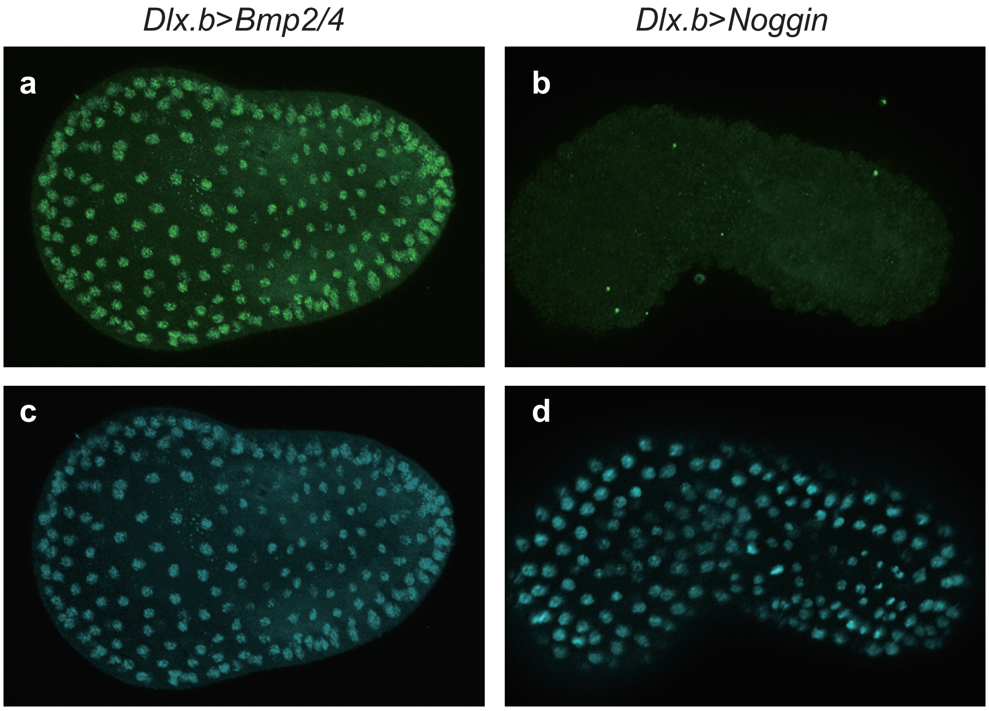

Supplementary Figure 2. The antibody used in this study successfully recognizes phosphorylated Smad1/5/8. (a) Antibody signal was detected in all epidermal cells of an embryo overexpressing *Bmp2/4* using the upstream sequence of *Dlx.b*. (b) No signal for phosphorylated Smad1/5/8 was detected in an embryo overexpressing *Noggin*. (c, d) The same embryos in (a) and (b) were stained with DAPI to detect nuclei. Photographs are Z-projected image stacks overlaid in pseudocolor.

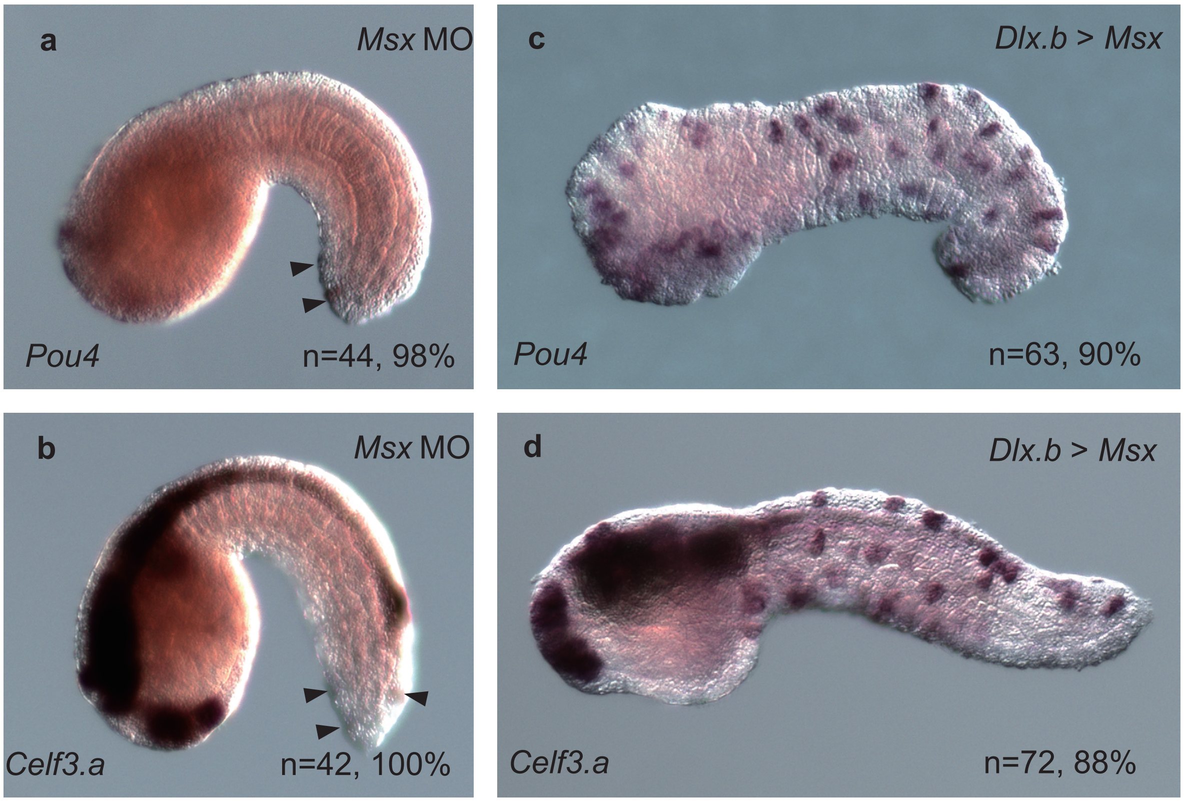

Supplementary Figure 3. Expression of *Pou4* and *Celf3.a* in dorsal and ventral ESNs is regulated by *Msx*. (a, b, c, d) Expression of *Pou4* (a, c) and *Celf3.a* (b, d), which marks ESNs, are downregulated in *Msx* morphants (a, b), except in a few cells near the tip of the tail, and upregulated in embryos with *Msx* overexpression. Arrowheads in (a) and (b) indicate cells that express *Pou4* and *Celf3.a*. Note that expression of *Pou4* and *Celf3.a* are not affected in embryos injected with *LacZ* MO, as shown in Fig. 7c, e. The number of embryos examined and the proportion of embryos that each panel represents are shown.

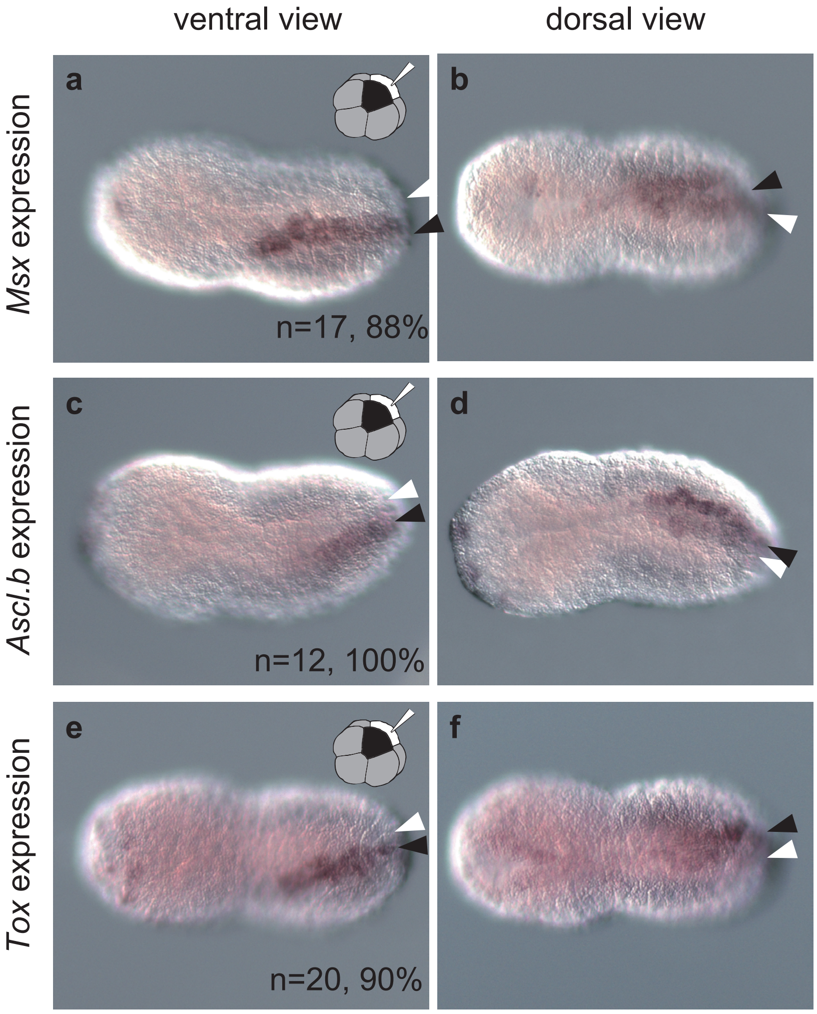

Supplementary Figure 4. Expression of *Msx*, *Ascl.b*, and *Tox* in ventral ectoderm is under the control of *Tbx2/3*. *Tbx2/3* MO was injected into the left posterior animal cell of 8-cell embryos (left b4.2; white cells in illustrations). (a, c, e) Ventral views of experimental embryos. Expression of (a) *Msx*, (c) *Ascl.b*, and (e) *Tox* in the ventral region was lost on the injected side (white arrowhead), but not on the uninjected side (black arrowhead). (b, d, f) Dorsal views of the embryos shown in (a), (c), and (e). Expression of (b) *Msx*, (d) *Ascl.b*, and (f) *Tox* in the dorsal region was not changed on the injected side (white arrowhead), or on the uninjected side (black arrowhead). The number of embryos examined and the proportion of embryos that each panel represents are shown. Injection of a control MO does not affect the expression of *Msx*, *Ascl.b*, and *Tox* (Fig.4i; Fig. 6ab).

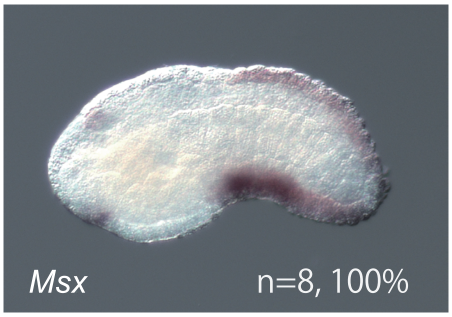

Supplementary Figure 5. Expression of *Tbx2/3* is not sufficient for activation of *Msx*. *Tbx2/3* mRNA was injected into both of the posterior animal cells at the 8-cell stage. The resultant embryos do not ectopically express *Msx* (compare the photograph with Fig. 3a).

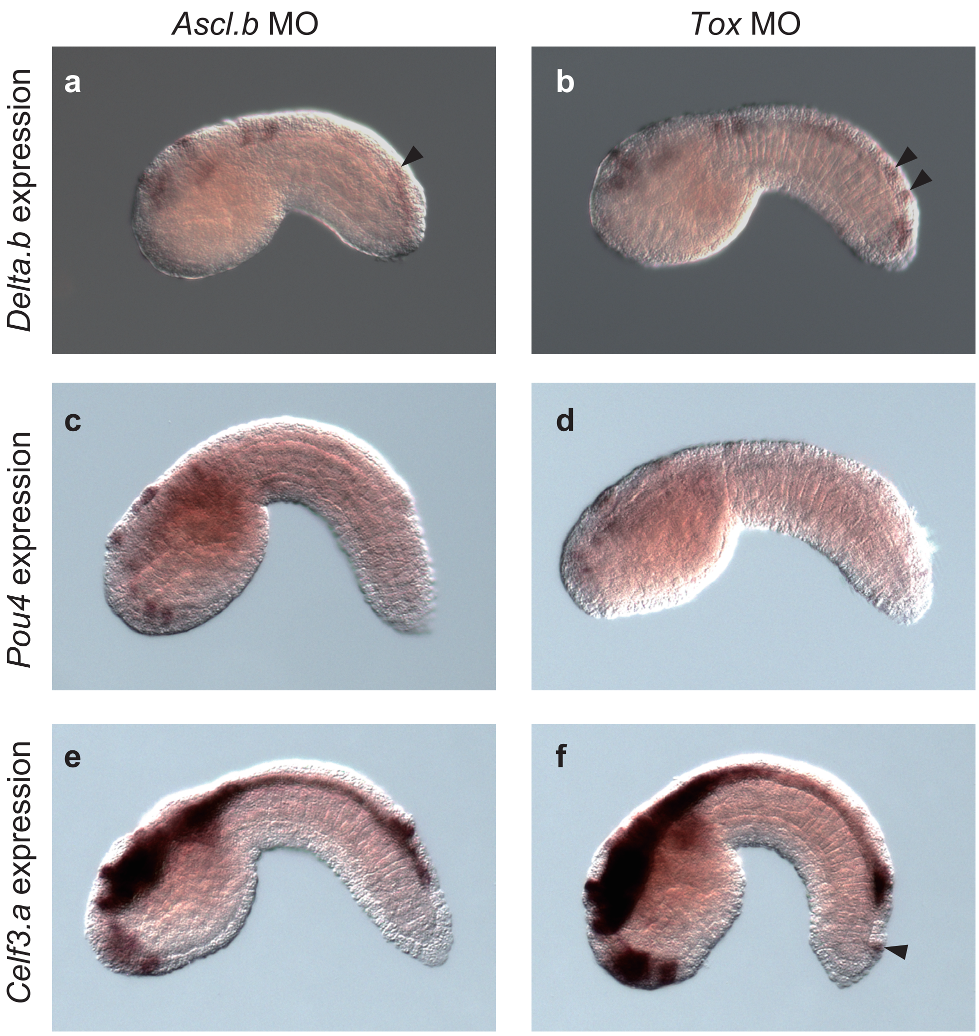

Supplementary Figure 6 Expression of *Delta.b*, *Pou4*, and *Celf3*.a is downregulated in morphants of *Ascl.b* and *Tox*. Expression of (a, b) *Delta.b,* (c, d) *Pou4*, and (e, f) *Celf3.a* is downregulated in (a, c, e) *Ascl.b* morphants and (b, d, f) *Tox* morphants. In some embryos, expression of *Delta.b*, *Pou4*, and *Celf3.a* is not completely lost, and several prospective ESNs express these genes (black arrowheads).

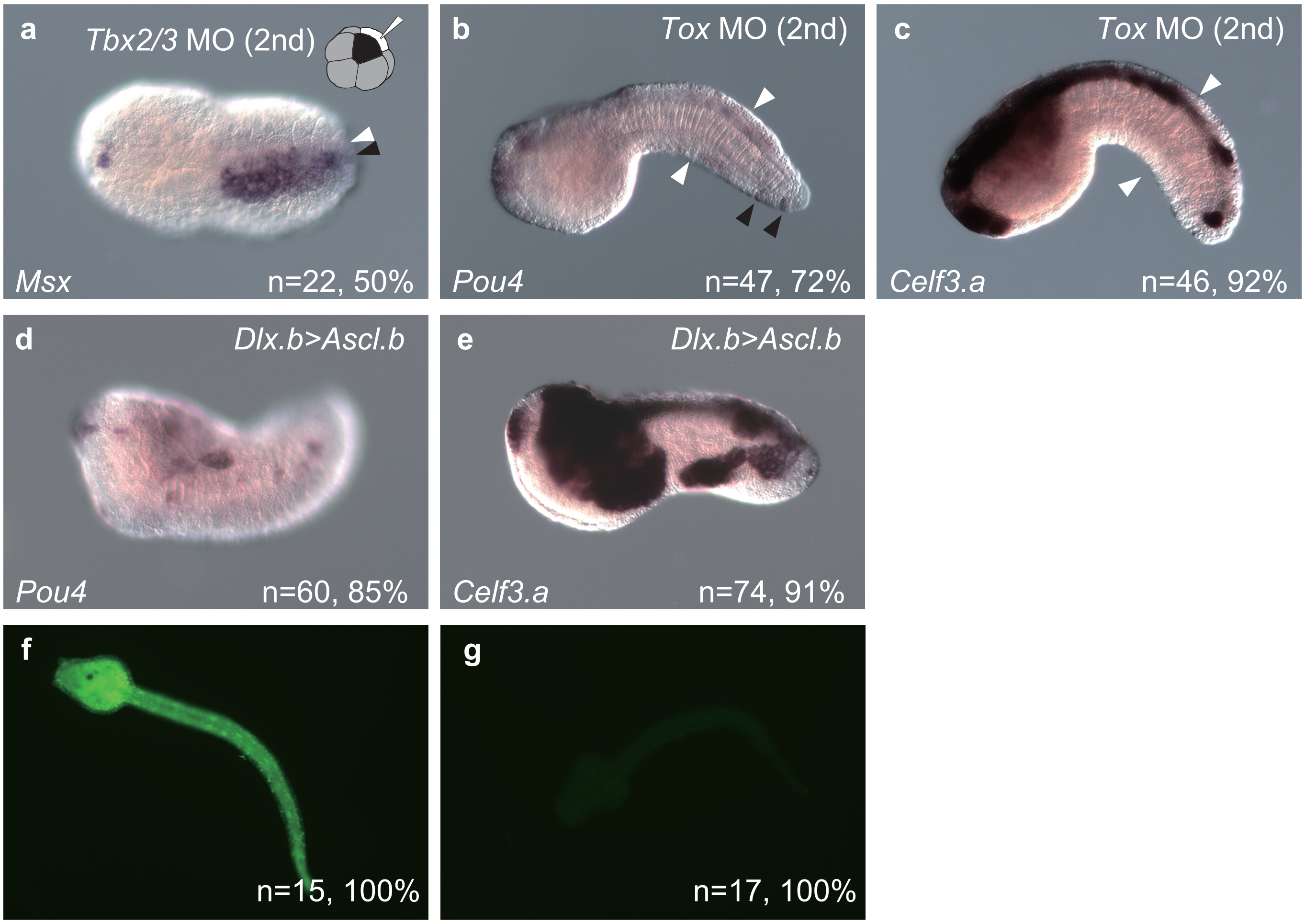

Supplementary Figure 7. Specific effects of morpholino oligonucleotides used in the present study. (a) When the second MO against *Tbx2/3* was injected into one b4.2 blastomere (a white cell in the top right illustration) at the 8-cell stage, *Msx* expression was lost on the injected side of the ventral ectoderm (white arrowheads) but not on the uninjected side (black arrowheads). A ventral view is shown. Both of the first and second MOs yielded the same phenotype (compare this photograph with Supplementary Fig. 4a). The number of embryos examined and the proportion of embryos that each panel represents are shown. (b, c) Expression of (b) *Pou4* and (c) *Celf3.a* is downregulated in morphant embryos injected with the second MO against for *Tox* (white arrowheads; compare (b) and (c) with Supplementary Fig. 6d, f). Lateral views are shown. Note that two or less cells occasionally expressed *Pou4* and *Celf3.a* in Tox morphant embryos (black arrowheads). The number of embryos examined and the proportion of embryos, in which *Pou4* and *Celf3.a* was expressed in a reduced number of cells, are shown. (d, e) The expression of (d) *Pou4* and (e) *Celf3.a* is seen in spots throughout the entire epidermal region of embryos with *Ascl.b* overexpression under the control of the upstream sequence of *Dlx.b* (compare (d) and (e) with Fig. 7c, e). Lateral views are shown. (f, g) Gfp expression in larvae injected with *Gfp* mRNA, in which the first codon was replaced with a nucleotide sequence including an *Ascl.b* MO target site. (f) Injection of *Gfp* mRNA alone resulted in strong Gfp expression, while (g) embryos co-injected with *Gfp* mRNA and the *Ascl.b* MO did not express Gfp. Photographs shown in (f) and (g) were taken with the same exposure time. The number of embryos examined and the proportion of embryos that each panel represents are shown.
